# Using landscape genomics to delineate seed and breeding zones for lodgepole pine

**DOI:** 10.1111/nph.18223

**Published:** 2022-06-13

**Authors:** Yue Yu, Sally N. Aitken, Loren H. Rieseberg, Tongli Wang

**Affiliations:** ^1^ Department of Forest Sciences, Centre for Forest Conservation Genetics University of British Columbia 3041‐2424 Main Mall Vancouver BC V6T 1Z4 Canada; ^2^ Department of Botany and Biodiversity Research Centre University of British Columbia 6270 University Boulevard Vancouver BC V6T 1Z4 Canada

**Keywords:** breeding zone, climate change, gradient forest, landscape genomics, local adaptation, lodgepole pine (*Pinus contorta*), seed zone

## Abstract

- Seed and breeding zones traditionally are delineated based on local adaptation of phenotypic traits associated with climate variables, an approach requiring long‐term field experiments. In this study, we applied a landscape genomics approach to delineate seed and breeding zones for lodgepole pine.
- We used a gradient forest (GF) model to select environment‐associated single nucleotide polymorphisms (SNPs) using three SNP datasets (full, neutral and candidate) and 20 climate variables for 1906 lodgepole pine (*Pinus contorta*) individuals in British Columbia and Alberta, Canada. The two GF models built with the full (28 954) and candidate (982) SNPs were compared.
- The GF models identified winter‐related climate as major climatic factors driving genomic patterns of lodgepole pine’s local adaptation. Based on the genomic gradients predicted by the full and candidate GF models, lodgepole pine distribution range in British Columbia and Alberta was delineated into six seed and breeding zones.
- Our approach is a novel and effective alternative to traditional common garden approaches for delineating seed and breeding zone, and could be applied to tree species lacking data from provenance trials or common garden experiments.

## Introduction

Local adaptation is a common phenomenon in widespread tree species, which results in among‐population genetic variation across the landscape (Morgenstern, 1996; Savolainen *et al*., 2007; Sork *et al*., 2016). Climate is a major environmental factor driving such variation (Morgenstern, 1996; Rehfeldt *et al*., 1999; Wang *et al*., 2010; Rehfeldt *et al*., 2014). Spatially explicit seed and breeding zones are widely used to account for local adaptation and among‐population variation when managing forest resources, such as with seed transfer guidelines (Ying & Yanchuk, 2006; Ukrainetz *et al*., 2018) to avoid the use of maladaptive seed sources for reforestation. Climate change induces a rapid shift of given habitat conditions and brings in new challenges to matching planting environments with the right seed sources. Thus, it has become necessary to develop climate‐based seed transfer models (O’Neill *et al*., 2017) to implement genetically informed management actions such as assisted gene flow (Aitken & Whitlock, 2013). Historically, seed or breeding zones divide the entire management area into a number of zones, and breeding materials and seed deployment are limited within each zone based on the ‘local is best’ rule (Morgenstern, 1996), which assumes that populations currently are well‐adapted to their local environment. However, with climate change, the ‘local climate’ is moving away, thus causing mismatch between the climate that tree populations have been adapted to and the climate that these populations will experience in the future (Aitken *et al*., 2008). Even with climate‐based seed transfer, similarly adapted populations need to be grouped to simplify management and deployment.

Variation in adaptive traits within management zones should be minimized to avoid maladaptation when populations are transferred within zones, while keeping the number of zones small to simplify management because it is expensive and operationally intractable to manage seed transfer and breeding strategies for each location independently, or for a large number of zones. Seed zone delineation is based on relationships between adaptive traits and climate variables that reflect the local adaptation of populations (Holst, 1962; Ying & Yanchuk, 2006). Such relationships conventionally are determined based on observations from long‐term common garden experiments (i.e. provenance tests), which are time‐consuming and expensive to establish and maintain. Also, long‐term common garden experiments are available mostly for commonly studied and economically important tree species such as lodgepole pine (*Pinus contorta* Dougl. Ex Loud.), Douglas‐fir (*Pseudotsuga menziesii* (Mirb.) Franco) and white spruce (*Picea glauca* (Moench) Voss; Howe *et al*., 2006; Liepe *et al*., 2016; Weng *et al*., 2018). With rapidly accumulating genomic data and resources in forest trees (Bragg *et al*., 2015), landscape genomic methods are emerging as an alternative for geographically defining populations for managing adaptive variation and tree breeding.

Landscape genomic approaches associate genomic variation rather than phenotypes from common garden experiments with environmental gradients (Savolainen, 2011; Sork *et al*., 2013). The gradient forest (GF) model is one method for establishing such an association. The GF model originally was developed to model spatial variation in community composition (Ellis *et al*., 2012), and was first used as a landscape genomics approach with single nucleotide polymorphism (SNP) data by Fitzpatrick & Keller (2015) to model turnover in allele frequencies along environmental gradients. Gradient forest uses the random forest machine learning approach (Breiman, 2001) to model the relationships between individual response variables (e.g. genomic information) and a multivariate set of predictors (e.g. climate variables), and aggregate these relationships into a nonlinear turnover function to transform multidimensional climate gradients into multidimensional biological (Ellis *et al*., 2012) or genomic gradients (Fitzpatrick & Keller, 2015; Jia *et al*., 2019). Simply put, a relationship is built between each SNP and climate variables using random forest, and GF aggregates those relationships across all selected SNPs. Therefore, feeding the GF model with climate gradients, we can predict overall genomic gradients.

Gradient forest has been successfully used to understand local adaptation and to guide conservation plans in balsam poplar (*Populus balsamifera*), Hawaii koa (*Acacia koa*) and oriental arborvitae (*Platycladus orientalis*) (Fitzpatrick & Keller, 2015; Gugger *et al*., 2018; Jia *et al*., 2019). As the GF model can predict the spatial pattern of genomic variation, it offers a new avenue to use genomic data to delineate seed and breeding zones, and to guide forest genetic resource management and conservation.

Lodgepole pine is a widespread forest tree species in western North America of considerable ecological and economic value. Previous studies of lodgepole pine have provided useful information on genecology (Wang *et al*., 2010; Gray *et al*., 2016; Mahony *et al*., 2020) and breeding zone delineation (Liepe *et al*., 2016; Ukrainetz *et al*., 2018). For example, Wang *et al*. (2006) showed that lodgepole pine populations may have a good performance over a range of environments and demonstrated that the ‘local is best’ rule is not always true. Efforts to delineate lodgepole pine seed and breeding zones attempt to balance high productivity and efficiency while minimizing the risk of maladaptation (Ukrainetz *et al*., 2018). To optimize such a delineation is important for breeding programs and seed deployments, and is constantly under exploration. For British Columbia (BC), historically, 16 breeding zones were mapped including different geographical areas and elevational bands for lodgepole pine (Snetsinger, 2005). In 2016, Liepe *et al*. conducted research on 2‐yr‐old seedlings in common garden experiments and suggested that such a large number of breeding zones was unnecessary. Instead, they reclassified populations in BC and Alberta (AB) into nine clusters. More recently, Ukrainetz *et al*. (2018) narrowed down the number of suggested breeding zones for BC alone into four based on genotype‐by‐environment (G × E) interactions through a Type‐B genetic correlation matrix using 28 lodgepole pine field‐based progeny tests. A high within‐group Type‐B genetic correlation means a low within‐group G × E interaction (Burdon, 1977).

These existing breeding zone delineations rely on phenotypic data from seedling experiments, field provenance trials or progeny tests to identify local adaptation patterns, which presents a barrier for many tree species that lack sufficient data or the resources for new field trials. The availability of SNP data from a large number of populations of lodgepole pine generated by the AdaptTree project (Yeaman *et al*., 2016) provided an opportunity to evaluate GF for delineating seed and breeding zones for this species.

In this study, we explored the gene–environment relationship and the spatial pattern of genomic composition in lodgepole pine over the western Canadian landscape using a GF model trained with three sets of SNPs. The results then were used to delineate seed and breeding zones covering British Columbia and Alberta, Canada. Our hypothesis was that the GF‐predicted multidimensional genomic gradients can adequately represent the underlying genomic basis of the locally adaptive traits. Thus, the seed and breeding zones delineated based on the GF model should be highly similar to those developed from phenotypic traits in provenance trials or common garden studies. If this hypothesis holds true, it suggests that this landscape genomics approach can be an effective alternative to long‐term field experiment‐based approaches for guiding seed and breeding zone delineation in forest management plans. Our specific objectives were to (1) identify major climate variables driving lodgepole pine local adaptation; (2) predict the spatial pattern of genomic composition over the landscape; (3) delineate lodgepole pine seed and breeding zones covering its distribution range in BC and AB; and (4) validate the performance of this genomic‐based approach for delineating breeding zones.

## Materials and Methods

### Study area

**Fig. 1:**
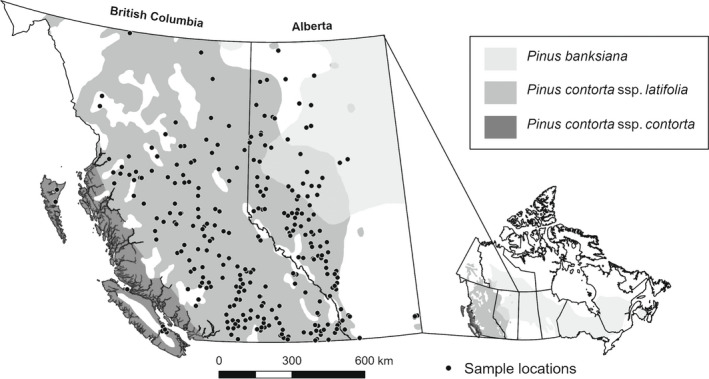
Distributions of lodgepole pine (*Pinus contorta*) subspecies and sample locations in British Columbia and Alberta, Canada. The smaller map on the right bottom is the map of Canada and its provincial borders. The geographical ranges of the coastal subspecies (*P. contorta* Dougl. Ex Loud. ssp. *contorta*), the interior subspecies (*P. contorta* Dougl. Ex Loud. ssp. *latifolia*), and overlapping areas with jack pine (*Pinus banksiana* Lamb.; Little Jr, 1971) were obtained from USGS (https://gec.cr.usgs.gov/data/little/, accessed 30 December 2020).

This study involved 281 lodgepole pine populations comprising 1906 individual seedlings grown from seed sampled across British Columbia (BC) and Alberta (AB) (Fig. 1). Selected provenances included the coastal subspecies (*P. contorta* ssp. *contorta*), the interior subspecies (*P. contorta* ssp. *latifolia*), as well as populations of the latter that are hybridizing with jack pine (*Pinus banksiana* Lamb.) (Mahony *et al*., 2020). The study area encompasses considerable climatic variation, ranging from 48.3°N to 60.0°N in latitude and 110°W to 149°W in longitude, and covers the main areas of productive lodgepole pine forests in western Canada. This study area also encompasses considerable climatic variation, with mean annual temperature ranging from –7.3°C to 10.3°C and mean annual precipitation ranging from 225 to 8769 mm.

### Genomic data

Single nucleotide polymorphism data of lodgepole pine were generated by the AdaptTree project (for more details, see https://adaptree.forestry.ubc.ca/) and methods are described in Mahony *et al*. (2020). Briefly, DNA was extracted from spring needle tissue using a Macherey‐Nagel Nucleospin 96 Plant II Core™ Kit (Fisher Scientific, Düren, Germany) on an EpMotion 5075™ liquid handling platform (Eppendorf, Hamburg, Germany). DNA samples were genotyped by Neogen GeneSeek with the AdapTree lodgepole pine Affymetrix Axiom 50K lodgepole pine SNP array designed from lodgepole pine sequence data (Suren *et al*., 2016; Yeaman *et al*., 2016). The SNP array included probes for the exons of genes, as well as intergenic regions, with intron–exon boundaries identified by mapping the lodgepole pine transcriptome to the loblolly pine (*Pinus taeda* L.) v.1.01 draft genome (Neale *et al*., 2014; Zimin *et al*., 2014). Candidate SNPs for climate adaptation were included on the array based on genotype–environment association (GEA) and genotype–phenotype association (GPA) analyses of exome capture data for the same populations (Suren *et al*., 2016; Yeaman *et al*., 2016). Genotypes for a total of 36 384 SNPs were obtained from the SNP array, from which three datasets were derived for this study (Mahony *et al*., 2020). The first dataset consisted of 32 407 SNPs, referred to as the ‘full set’ (MacLachlan *et al*., 2017; Mahony *et al*., 2020, SNP data available at: doi: 10.5061/dryad.56j8vq8). The second dataset consisted of the mentioned 3934 intergenic SNPs to control for neutral population structure, referred to as the ‘neutral set’, which had no overlap with the full set (also available at: doi: 10.5061/dryad.56j8vq8). The third dataset consisted of 1172 SNPs selected from the full set by both GEA using bayenv2 (Coop *et al*., 2010) and GPA by selecting the bottom 1% of GPA *P*‐values (one SNP per contig) for four traits: height, cold injury, growth initiation and growth cessation (Mahony *et al*., 2020; MacLachlan *et al*., 2021). This dataset was referred to as the ‘candidate set’, and it included 864 SNPs selected by the GEA analysis and 513 unique SNPs filtered by the GPA analysis (Mahony *et al*., 2020; MacLachlan *et al*., 2021). After excluding 205 overlapping SNPs in both the GEA and the unique GPA set, we retained a total of 1172 SNPs as the ‘candidate set’. The candidate set was a subset of the full set, but the neutral set was not included in the full set.

All three datasets were further selected with minor allele frequencies among all individuals > 0.05, because rare alleles are likely to cause false positives in further analysis (Gugger *et al*., 2018; Ruegg *et al*., 2018). Therefore, a final number of 28 954 SNPs for the ‘full set’, 3580 SNPs for the ‘neutral set’ and 982 SNPs for the ‘candidate set’ were used to build gradient forest (GF) models. The GF models built with the three SNP datasets are referred to as the ‘full model’, ‘neutral model’ and ‘candidate model’, respectively.

### Climate variables

Climate variables were obtained using climatena software (Wang *et al*., 2006, 2016). climatena extracted and downscaled gridded (800 m × 800 m) climate data from Prism (Daly *et al*., 2008) to scale‐free specific locations through dynamic local downscaling (Wang *et al*., 2016). For GF model development, 20 annual climate variables (Table 1) were generated for the 281 sampling locations for the 1961–1990 reference period. For spatial predictions, a digital elevation model (DEM) input raster file covering the study area was cropped from the DEM raster input file (800 m × 800 m) for North America included in the climatena package (v.6.30), and the spatial climate variables were generated in raster format for the 1961–1990 reference period.

**Table 1:** List of the 20 climate variables from climatena for this study and a brief description of each.

Abbreviation	Variable description
Eight annual variables calculated from monthly climate variables	
MAT	Mean annual temperature (°C)
MWMT	Mean warmest month temperature (°C)
MCMT	Mean coldest month temperature (°C)
TD	Temperature difference between MWMT and MCMT, or continentality (°C)
MAP	Mean annual precipitation (mm)
MSP	Mean annual summer (May to Sept.) precipitation (mm)
AHM	Annual heat‐moisture index (MAT + 10)/(MAP/1000))
SHM	Summer heat‐moisture index ((MWMT)/(MSP/1000))
12 annual variables derived from monthly climate variables	
DD < 0	Degree‐days below 0°C, chilling degree‐days
DD > 5	Degree‐days above 5°C, growing degree‐days
NFFD	Number of frost‐free days
FFP	Frost‐free period
bFFP	Day of the year on which FFP begins
eFFP	Day of the year on which FFP ends
PAS	Precipitation as snow (mm). For individual years, it covers the period between August in the previous year and July in the current year
EMT	Extreme minimum temperature over 30 yr
EXT	Extreme maximum temperature over 30 yr
Eref	Hargreaves reference evaporation (mm)
CMD	Hargreaves climatic moisture deficit (mm)
RH	Mean annual relative humidity (%)

### Gradient forest model

Genotypes for each SNP were used as the dependent variables and the 20 annual climate variables obtained from climatena were used as predictor variables for model fitting. The GF model was built using the gradientforest package in R v.3.5.1 (Ellis *et al*., 2012; Gugger *et al*., 2018; R Core Team, 2018) with 200 trees as suggested by Oshiro *et al*. (2012), and all other parameters at default settings (Ellis *et al*., 2012). For more details of the gradient forest model, see Supporting Information Methods S1. The trained GF model then was used to predict the genomic variation for the distribution of lodgepole pine within the study areas (including both the coastal and interior subspecies range shown in Fig. 1) based on the spatial climate variables in raster format (using R/raster) (Hijmans, 2021). The GF predictions represent the multidimensional genomic variation of lodgepole pine across its distribution in BC and AB, Canada.

### Spatial genomic variation

After GF model prediction, we performed principal components analysis (PCA) using the default stats package in R to summarize the GF‐predicted multidimensional genomic variation of lodgepole pine. The first two dimensions are presented in a biplot. Following Fitzpatrick & Keller (2015), the top three principal components (PCs) were used to define the RGB color palette for the biplot. Similar colors in the biplot space represented similar expected genomic composition. The biplot also included loadings of all 20 environmental predictors, which explained the magnitude and direction of data distribution. We further mapped the PC scores based on geographical coordinates to present the continuous change in genomic variation of lodgepole pine over the study area, referred to as the continuous map of GF‐predicted genomic variation.

### Seed and breeding zone delineation

Principal components analysis results representing the GF‐predicted continuous genomic variation then can be clustered into different numbers of classes to define populations or to delineate seed and breeding zones. We used the partitioning around medoids clustering method and R/factoextra (Kassambara & Mundt, 2020) to calculate the within‐cluster variation for different numbers of clusters based on the GF‐predicted continuous genomic variation. We explored cluster numbers varying from 2 to 16, the latter being the initial number of breeding zones originally used for lodgepole pine in BC (Snetsinger, 2005). The optimal number of clusters was determined by examining results for the point at which adding one more cluster explains substantially less within‐cluster variation than adding previous clusters did, termed the ‘elbow point’ (Kassambara, 2017). The selected number of clusters was used to group the spatial distribution of the continuous GF‐predicted genomic variation into GF‐based seed and breeding zones for the study area.

### Seed and breeding zone comparisons

We applied two comparisons to validate the effectiveness of this genomic‐based approach for zone delineation. First, we compared our proposed optimal number of GF‐based zones with the two exsiting seed and breeding zones delineated based on phenotypic data by Liepe *et al*. (nine zones for BC and AB) and Ukrainetz *et al*. (four zones for BC), referred to as ‘common garden‐based seed and breeding zones’ hereafter. This comparison intended to spatially quantify the proportion of the existing zones matching our optimal GF‐based zones and is termed a ‘forward comparison.’ Liepe *et al*. (2016) delineated nine adaptive zones covering the range of 281 lodgepole pine populations within BC and AB, including the same BC populations analyzed in this study (Figs 1, 4), but Liepe *et al*.’s delineation was based on a combination of seedling adaptive traits and provincial ecosystem classifications. Ukrainetz *et al*. (2018) delineated BC into four seed and breeding groups based on Type‐B genetic correlations among sites using field progeny test data for south and central BC. In our second comparison, we adjusted the GF‐based delineations into four and nine zones to match the two existing delineations, respectively. This comparison was to determine the ability of the GF‐based approach in reproducing the two existing delineations, thus referred to as ‘backward comparison.’

Shapefiles of both the GF‐based seed and breeding zones and the two existing zone maps were imported into *ArcGIS* to perform spatial comparisons (in area). Overlapping areas were identified using the *clip* tool with the common garden‐based zones as the input feature and the GF‐based zones as the clip feature. All zone areas were calculated using the *summary statistics* tool in square kilometers. The final averaged overlap rate in each comparison was calculated as the percentage of the overlapping areas.

## Results

### GF models with different SNP sets

With the full SNP dataset, the GF model selected 402 SNPs with positive predictive power, out of the total of 28 954 SNPs (1.4%), to build the final model (Methods S1; Table 2). A much higher proportion of SNPs with positive predictive power (19.7%, 193 of 982 SNPs) were selected from the candidate set in building the second GF model. Of the 193 selected SNPs, 98% of them (189 SNPs) overlapped with the SNPs selected from the full set. By contrast, only 0.1% (two of 3580) SNPs were selected from the neutral set by the GF model. The contrasting proportions of the SNPs selected by the GF model between the candidate set and the neutral set suggest the good potential of GF models to identify locally adaptive SNPs. The mean *R* ^2^ value, an analogous measure for the deviance explained by each SNP in GF model (Leaper *et al*., 2011), was the highest for SNPs in the GF model for the full set, closely followed by the ones for the candidate set. By contrast, the GF model for the neutral set had a much lower mean *R* ^2^ value. Thus, we eliminated the neutral set for further analysis.

**Table 2:** Summary of the three single nucleotide polymorphism (SNP) datasets used for gradient forest (GF) model fitting and the performance of each GF model.

SNP dataset	SNPs before filtering	SNPs with MAF > 0.05	SNPs with positive *R* ^2^ (%)	Overlap with the full set	Mean *R* ^2^ (min, max) (%)*
Full	32 407	28 954	402 (1.4)	402	2.7 (0.0, 55.9)
Neutral	3934	3580	2 (0.1)	0	1.0 (0.2, 4.0)
Candidate	1172	982	193 (19.7)	189	2.6 (0.0, 52.4)

* Mean *R* ^2^ is the mean calculated among all SNPs with a positive *R* ^2^ value (Supporting Information Methods S1). MAF, minor allele frequency.

### From environmental gradients into biological gradients

**Fig. 2:**
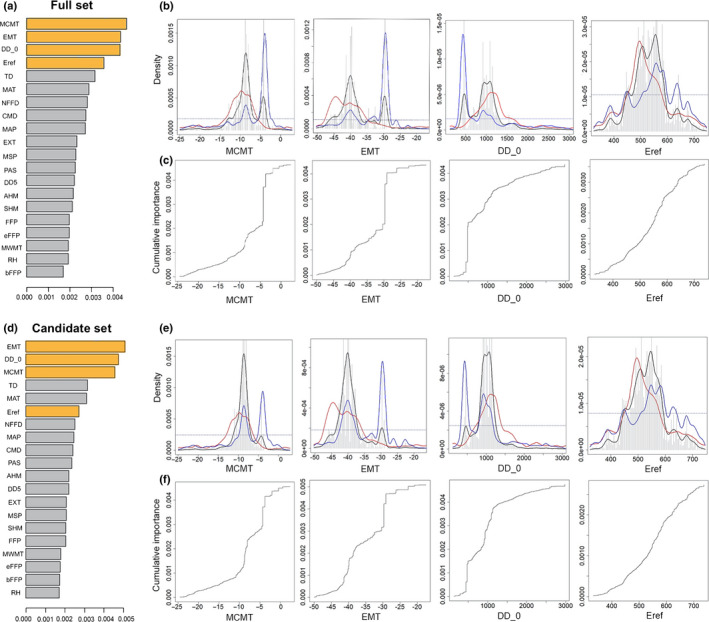
The gradient forest (GF) model output for the full and the candidate single nucleotide polymorphism (SNP) sets for lodgepole pine (*Pinus contorta*). Overall importance of all 20 climate predictors for the full set (a) and for the candidate set (d). Importance values for each climate variable are in Supporting Information Table S1. The four most important climate variables for the full GF model are highlighted in yellow and include three variables related to low temperatures and the top moisture‐related variable (Hargreaves reference evaporation (Eref)). These variables also are highlighted for the candidate set and are further presented in (b) and (c) for the full set and in (e) and (f) for the candidate set. Split‐density graph of the highlighted climate predictors for the full set (b) and for the candidate set (e): mean coldest month temperature (MCMT), extreme minimum temperature (EMT) over 30 yr, degree‐days (DD) below zero degrees (DD < 0, presented as DD_0 in graphs) and Eref. For split‐density graphs of all predictors, see Figs S1 and S2 for the full and candidate model. Gray bars, binned raw importance density generated by the random forest output; black line, raw importance density *I*(*x*); red line, density of data *d*(*x*); blue line, estimated importance *f*(*x*) = *I*(*x*)/*d*(*x*); dashed horizontal line, where the *f*(*x*) ratio is one (Ellis *et al*., 2012). Predictor cumulative importance curves for the four highlighted predictors are presented (MCMT, EMT, DD_0 and Eref) for the full set (c) and for the candidate set (f). For all predictors, see Figs S3 and S4 for the full and candidate model.

The GF model associates the changes in allelic composition of the selected SNPs along climatic gradients and transforms the climatic gradients into genomic gradients. The importance of the 20 climate variables, as explained by *R* ^2^ values with predictive SNPs, varied from 0.0017 to 0.0046 for the full set (Fig. 2a; Table S1) and from 0.0017 to 0.0051 for the candidate set (Fig. 2d; Table S1). The top three climate variables in terms of importance identified both by the full and candidate set were the same, including mean coldest month temperature (MCMT), degree‐days below zero (DD < 0), and extreme minimum temperature over 30 yr (EMT). The fourth climate variable is Hargreaves reference evaporation (Eref) in the full model and continentality (TD) in the candidate model, in which Eref ranked sixth. The first three are related to minimum winter temperatures, whereas Eref is a moisture‐related climate variable.

The combined importance across all of the predictive SNPs, indicating allelic change along an environmental gradient, is represented by the blue line for each climate variable (Fig. 2b,e). This blue line is the estimated importance *f*(*x*), calculated as raw importance density *I*(*x*) (black line) divided by the density of data *d*(*x*) (red line). When the blue line was above the dashed line (the turnover rate at 1, base turnover rate line), an equal amount of predictor range change would result in a higher allelic change. For the full set, significant allelic change occurred between −9°C and −2°C for MCMT, between −42°C and −38°C as well as between −35°C and −28°C for EMT, between 300 and 1200 for DD < 0, and between 500 and 600 mm for Eref, respectively (Fig. 2b). Similar ranges were found in the candidate set for the top‐ranked climate variables (Fig. 2e).

The cumulative importance curve (turnover curve), as the integral of estimated importance, reveals genomic variation compositional changes along each predictor gradient. The ranges of a predictor with high turnovers in the cumulative importance curve were associated with the range of significant allelic change shown in the estimated importance graph (Fig. 2b,c,e,f). For example, several turnover points occurred between −42°C and −38°C and between −35°C and −28°C for EMT, suggesting a simultaneous, rapid change in allelic composition for multiple loci among lodgepole pine populations.

### Spatial genomic variation

**Fig. 3:**
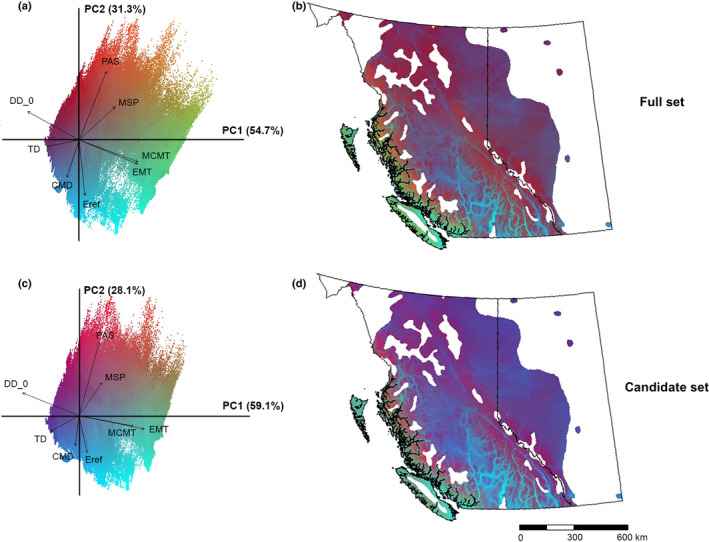
Genomic variation of lodgepole pine (*Pinus contorta*) predicted by the full set (a, b) and by the candidate set (c, d) across British Columbia (BC) and Alberta (AB), Canada. Similar colors in the sampled space correspond to similar expected genetic composition associated with climate. (a) Principal component analysis (PCA) biplot of the genomic variation based on principal components (PCs), PC1 (accounts for 54.7% of the variation) and PC2 (31.3%) for the full set. Each point in the biplot represents a location in the lodgepole pine distribution range in BC and AB. Vectors show eight environmental predictors (CMD, Hargreaves climatic moisture deficit; DD_0, degree‐days below 0 degrees; EMT, extreme minimum temperature over 30 yr; Eref, hargreaves reference evaporation; MCMT, mean coldest month temperature; MSP, mean annual summer precipitation; PAS, precipitation as snow; TD, continentality). For vectors of all climate variables, see Supporting Information Fig. S5. (b) Spatial distribution of the gradient forest (GF)‐predicted genomic variation for the full set. (c) Principal components analysis biplot of the genomic variation based on PC1 (59.1%) and PC2 (28.1%) for the candidate set; for vectors of all climate variables, also see Fig. S5. (d) Spatial distribution of the GF‐predicted genomic variation for the candidate set.

The first three principal components of the transformed multidimensional genomic gradients accounted for 91.2% and 92.2% of the variation in SNP allelic composition for the full and the candidate set cumulatively, respectively. The spatial pattern of genomic variation represented by the first three PCs is very similar between the full and the candidate set. Results from both sets showed a clear genomic composition difference in lodgepole pine populations between the coastal and the interior regions (Fig. 3b,d). In general, the distribution of genomic variation among coastal populations was associated with winter temperature (MCMT and EMT in Fig. 3a,c) and summer precipitation (MSP), whereas genomic variation in the interior populations in central and southern interior BC was associated with indices that combine moisture and temperature to calculate moisture deficits (CMD and Eref). Genomic variation among populations also was observed along elevational gradients across the study area. Populations residing in high‐elevation regions (Fig. S6) were affected by precipitation, including precipitation as snow (PAS) and summer precipitation (MSP). Valley populations were influenced mainly by moisture deficits (CMD and Eref).

### Estimation of the number of seed and breeding zones

**Fig. 4:**
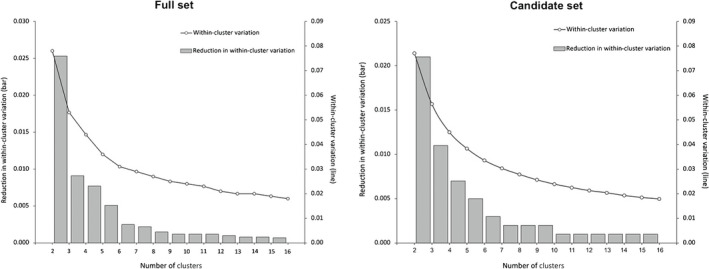
Within‐cluster variation (black solid line) and its reduction (gray bars) with the increase in the number of clusters estimated from the lodgepole pine (*Pinus contorta*) gradient forest (GF)‐predicted continuous genomic variation by the full set (left) and the candidate set (right).

Within‐cluster variation estimated from the GF‐predicted genomic gradients decreased gradually from 2 to 16 clusters. The pattern of decrease was similar between the two models, declining from 0.078 at two groups to 0.018 at 16 groups for the full set, and from 0.077 to 0.018 for the candidate set, respectively (Fig. 4; Table S2).

This decrease followed a relatively smooth trend (black solid line; Fig. 4), and therefore no clear elbow point could be identified. However, the reduction in within‐cluster variation (gray bars; Fig. 4) showed that six clusters for the full set and six or seven clusters for the candidate set were the minimum number of clusters meeting the condition that adding one more cluster explains substantially less within‐cluster variation than adding previous clusters did. Although additional clusters would further reduce within‐cluster variation, a larger number of seed or breeding zones also would increase the complexity of seed resource management. Therefore, we used six clusters for the final GF‐based seed and breeding zone delineation for both SNP sets for downstream comparisons.

### GF‐based seed and breeding zone delineation

**Fig. 5:**
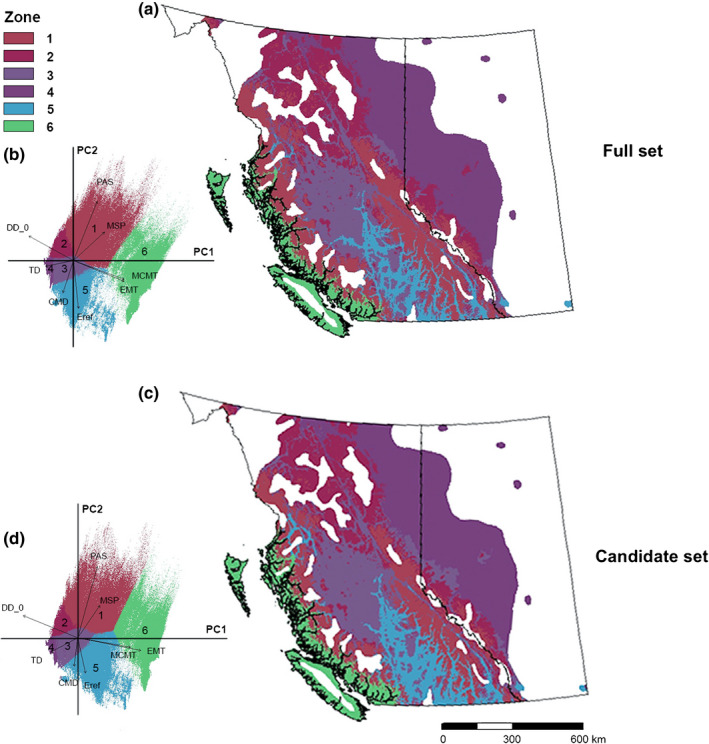
Six seed and breeding zones based on the full and the candidate gradient forest (GF) models for lodgepole pine (*Pinus contorta*) in British Columbia (BC) and Alberta (AB), Canada. (a) Spatial distribution of the six seed and breeding zones based on the full GF model. (b) Principal component analysis (PCA) biplot of the full GF‐predicted genomic variation showing the six clusters used for seed and breeding zone delineation. Principal component (PC)1 and PC2 account for 54.7% and 31.3% of the variation, respectively. Each point in the biplot represents a location in the lodgepole pine distribution range in BC and AB. Vectors show eight environmental predictors that explain the data distribution (CMD, hargreaves climatic moisture deficit; DD_0, degree‐days below 0 degrees; EMT, extreme minimum temperature over 30 yr; Eref, hargreaves reference evaporation; MCMT, mean coldest month temperature; MSP, mean annual summer precipitation; PAS, precipitation as snow; TD, continentality). (c) Spatial distribution of the six seed and breeding zones based on the candidate GF model (colors in (c) were adjusted to the six seed and breeding zones based on the full GF model to facilitate comparison with (a)). (d) Principal components analysis biplot of the candidate GF‐predicted genomic variation. PC1 and PC2 account for 59.1% and 28.1% of the variation, respectively.

The six GF‐based seed and breeding zones for lodgepole pine in BC and AB generated by the full and candidate set shared a highly similar pattern (Fig. 5). In both GF‐based delineations, colors in corresponding zones were matched to facilitate comparisons. Zone 6 occurred exclusively in BC and the other five were shared by the two provinces. Zone 6 covered areas of low‐ to mid‐elevation (< 1500 m above sea level) and subalpine areas along the Coast Mountains (Fig. S6). This zone was associated with the coastal climate (Fig. 5). High elevation areas (1500–2000 m asl) of the lodgepole pine range in the interior of BC and AB were classified into Zones 1 and 2. Within BC, Zone 1 was distributed in the Coast Mountains and Columbia Mountain region (Fig. S6), Zone 2 in the Bulkley–Skeena region of northwestern BC, and the two zones (1 and 2) together occupied the Eastern Continental Ranges in AB. Zone 5 was heavily influenced by moisture‐ and drought‐related climate variables (Fig. 5) as it covered the mid‐elevation (1000–1500 m asl) areas of Cariboo–Chilcotin, Thompson–Okanagan, Kootenay and Bulkley–Skeena regions. However, the low elevation (500–1000 m asl) areas of these regions were classified into Zone 3. Zone 4 was distributed mainly in the Foothills and Boreal Forest natural regions of western AB and covered portions of the Peace River region of BC.

### Comparisons between GF‐based and common garden‐based seed and breeding zones

In the forward comparison, the six seed and breeding zones based on the the full GF model shared average overlap rates of 68.6% and 63.6% in area with the common garden‐based seed and breeding zones proposed by Liepe *et al*. (2016) and by Ukrainetz *et al*. (2018), respectively (Table 3). The corresponding average overlap rates for the candidate GF model were slightly lower (67.3% and 60.8%, repectively) but varied among the zones. In the comparison with the nine zones by Liepe *et al*., high matching rates occurred between GF‐based Zone 6 and Liepe *et al*.’s Zone 9 (Coastal BC), with matching rates of 71.9% for the full model and 73.1% for the candidate model. Matching rates were poorer between GF‐based Zone 1 and Liepe *et al*.’s Zone 3 (Montane BC; Table 3) with a rate of 49.0% for the full model and 43.0% for the candidate model. Other zones mostly had a similar matching rate between the full and candidate model, with an exception between GF‐based Zone 5 and Liepe *et al*.’s Zone 8 (Interior Valleys). The candidate model in this case showed a higher matching rate of 81.9% compared to 65.7% for the full model, because the candidate model captured a larger range within the Northwest Coast region into GF‐based Zone 5, which aligned better with Liepe *et al*.’s Zone 8.

**Table 3:** Forward comparisons between the six seed and breeding zones delineated based on the full and the candidate gradient forest (GF) models with two sets of common garden‐based seed and breeding zones delineated previously by Liepe *et al.* (2016) and by Ukrainetz *et al.* (2018) for lodgepole pine in western Canada.

Common garden‐based zones	GF‐based zone number	Common garden‐based zone (zone number)	Full set			Candidate set		
			Overlap rate (%)	Overlapping area (km^2^)	Averaged overlap rate (%)	Overlap rate (%)	Overlapping area (km^2^)	Averaged overlap rate (%)
Liepe *et al*. (2016) zones	1	Montane BC (3)	49.0	22 784	68.6	43.0	19 978	67.3
	2 and 4	Montane AB (1), Lower Foothills (2), Lower Boreal Highlands AB (4) and Dry Mixed Wood AB (5)	70.2	255 503		67.5	245 840	
	3	Sub‐boreal (6 and 7)	69.0	121 821		66.4	117 284	
	5	Interior Valleys (8)	65.7	23 293		81.9	29 042	
	6	Coastal BC (9)	71.9	17 662		73.1	17 961	
Ukrainetz *et al*. (2018) zones	1 and 2	Breeding group 4	70.4	309 948	63.6	63.5	279 711	60.8
	3 and 4	Breeding group 3	73.9	177 476		75.4	180 914	
	5	Breeding group 2	36.0	52 629		46.0	67 289	
	6	Breeding group 1	25.0	64 950		28.7	74 490	

Liepe *et al*. (2016) delineated British Columbia (BC) and Alberta into nine zones, whereas Ukrainetz *et al*. (2018) had four zones for BC. Average overlap rates were calculated as the percentage of the areas in a zone that overlapped.

The four breeding zones delineated by Ukrainetz *et al*. (2018) cover a smaller geographical region than the current study, and when compared with the six GF‐based zones, a high overlap occurred between GF‐based zones 3 and 4 (Fig. 5) and Ukrainetz *et al*.’s breeding group 3 (fig. 6 in Ukrainetz *et al*., 2018), with a matching rate of 73.9% for the full model and 75.4% for the candidate model. A moderate overlap (70.4% for the full model and 63.5% for the candidate model) was found between GF‐based zones 1 and 2 and Ukrainetz *et al*.’s breeding group 4. The remaining zones had low overlap rates (< 50%).

The forward comparison had much higher overlap rates than the backward comparison between the GF‐based zones and the two common garden‐based delineations for both of the full and candidate GF models. For the backward comparison, nine and four zones were delineated based on the GF models (Fig. S7) and compared to the nine zones by Liepe *et al*. (2016) and the four zones by Ukrainetz *et al*. (2018), respectively. The average overlap rates with the nine zones were 49.5% and 53.5% for the full and the candidate model, respectively. The corresponding overlap rates with the four zones were slightly higher (56.8% and 57.4%, respectively) (Table S3). In both cases, the overlap rates were slightly higher for the candidate model. Likewise, the overlap rates varied among zones.

## Discussion

An effective delineation of seed or breeding zones should reflect patterns of among‐population locally adaptive variation for targeted forest tree species, and ensure that genetic materials from each zone can be deployed within the same range of environmental conditions for optimal adaptation and productivity with acceptable risks (Holst, 1962; Li *et al*., 2017). Traditionally, the delineation of seed or breeding zones was achieved based on observations from long‐term field experiments, including provenance (Ying & Yanchuk, 2006) and progeny tests (Ukrainetz *et al*., 2018). Our study applied a landscape genomics approach and built gradient forest (GF) models using two SNP datasets and climate variables for lodgepole pine in two western Canadian provinces (British Columbia and Alberta). Our results showed that the GF models were able to select environment‐associated SNPs and transform the multidimensional climate gradients into multidimensional genomic gradients. Based on the GF model predictions, we delineated the lodgepole pine distribution range in British Columbia (BC) and Alberta (AB) into six seed and breeding zones. We further validated our approach by comparing the genomic‐based delineations with the previous common garden‐based delineations, and found a relatively high overlap in area. To the best of our knowledge, this is the first application of landscape genomics to delineate region‐wide seed and breeding zones with validation.

### Climate variables driving genomic variation

The climate variables identified as important for predicting phenotypic and genomic variation are strongly correlated in lodgepole pine (Mahony *et al*., 2020), with winter temperature‐related climate variables consistently found to be the major drivers of among‐population variation. Our study also confirmed three winter temperature‐related variables (mean coldest month temperature (MCMT), extreme minimum temperature over 30 yr (EMT) and degree‐days below zero (DD < 0)) as the most important climate variables. In addition, we identified moisture‐deficit related variables (Eref) also playing an important role in shaping the genomic variation of this species, especially between lodgepole pine populations in coastal and interior regions.

### Transformation of climate gradients into biological gradients

The GF model can effectively predict genomic gradients along climate gradients (Fitzpatrick & Keller, 2015). Our study found that the GF‐predicted continuous genomic variation aligned well with the macroclimatic patterns in the region (Fig. 3b,d). For example, the map of allelic composition clearly distinguished the coastal and interior regions, which matched the distribution of two subspecies of lodgepole pine: the coastal subspecies (ssp. *contorta*) and the interior subspecies (ssp. *latifolia*). In addition, the allelic composition (Fig. 3b,d) showed some similarities to the pattern of phenotypic variation predicted based on the provenance trials of this species in BC (fig. 4a in Wang *et al*., 2010), supporting the potential of the GF‐predicted genomic variation to reflect the local adaptation of populations and serving as the basis for the delineation of seed and breeding zones for populations.

### Effectiveness of seed and breeding zone delineation

The principle of delineating seed or breeding zones is to group areas with similar climate conditions and populations with similar adaptive genotypes so that the genetic materials from the same zone can be deployed anywhere within a zone with a low risk of maladaptation. Additionally, the number of zones should be kept reasonably small to simplify management. Although species like lodgepole pine that are continuously distributed would not be expected to have discretely distributed adaptive variation underlying local adaptation, it is helpful to define regional clusters or zones comprising similarly adapted provenances for management and breeding. The relationship between adaptive traits and climate variables has been the standard basis for delineating seed or breeding zones. Such relationships for lodgepole pine have been determined based on adaptive traits observed from common‐garden experiments in the field (Ying & Yanchuk, 2006; Ukrainetz *et al*., 2018) and in a seedling common garden experiment in growth chambers (Liepe *et al*., 2016). In this study, we applied genomic data to achieve such an objective using GF models. However, to understand the effectiveness of our approach, it is critical to compare our genomic‐based seed and breeding zones with zones previously delineated based on common garden observations.

We conducted forward comparisons to quantify the proportions of the existing zones spatially represented by the GF‐based zones and backward comparisons to determine the ability of the GF‐models to reproduce the existing common garden‐based zones. On the one hand, we found relatively high overlap rates with both Liepe *et al*.’s nine zones (68.6% for the full model and 67.3% for the candidate model on average; Table 3) and Ukrainetz *et al*.’s four zones (63.6% for the full model and 60.8% for the candidate model on average; Table 3) in the forward comparisons, suggesting the effectiveness of the six GF‐based zones. On the other, the backward comparisons showed only moderate overlap rates with Liepe *et al*.’s nine zones (49.5% for the full model and 53.5% for the candidate model on average; Table S3) and with Ukrainetz *et al*.’s four zones (56.8% for the full model and 57.4% for the candidate model on average; Table S3).

The higher overlap rates found in our forward comparisons were expected. This was because the six zones were identified to be the optimal number of zones to delineate the entire study area based on the spatial genomic variation (Fig. 4). The differences were greater when the number of zones was increased from six to nine for the comparisons with Liepe *et al*.’s zones than when the number of zones was decreased from six to four for the comparisons with Ukrainetz *et al*.’s zones. This also is reasonable as more zones reflecting more fine‐scale details are more likely to show differences between the two approaches. The combination of the forward and backward comparisons suggests the importance of using the optimal number of zones; an increase or decrease from the optimal number of zones would compromise the effectiveness of the GF‐based delineations.

In addition, our forward comparison using the GF‐based six zones with Ukrainetz *et al*.’s delineation of four zones suggests two sensible improvements in seed and breeding zone delineation for lodgepole pine in western Canada. First, separating the coastal regions from the interior in our GF‐based zones is biologically appropriate based on the large ecological and climatic differences between coastal and interior areas, as well as the variation between subspecies *contorta* and *latifolia*. Second, Ukrainetz *et al*. (2018) indicated that breeding group 4 covering a large area in northern BC probably requires refinement due to model classification errors. Our GF‐based zones divided breeding group 4 into two zones (Fig. 5, GF‐Zone 1 and GF‐Zone 2), which resulted in a better alignment with the BEC zones in Engelmann Spruce–Subalpine Fir (ESSF) and Spruce–Willow–Birch (SWB). Low latitude, high elevation and subalpine boreal climates are the typical characteristics of the ESSF zone. Thus, it is reasonable for it to be separated from the SWB zone, which occupies the middle elevations of northern interior BC with less precipitation and lower mean annual temperature (Meidinger & Pojar, 1991; Table S4).

Overall, comparisons with the common garden‐based zones support the effectiveness of the GF‐based seed and breeding zone delineation using genomic data. Delineation accuracy depends on the effective representation of genomic variation over the landscape by the GF model predictions. A major concern is its ability to detect local adaptation rather than neutral population structure. Given the extremely low SNP selection rate on the neutral set (Table 2), the GF model appears to have included very few false‐positive SNPs not associated with the environment. It appears that the spatial pattern of genetic variation predicted by the GF model is not significantly affected by population structure in this case, and the delineation of seed and breeding zones using the GF model is likely feasible to other widespread forest trees species with limited population structure.

### Conclusion

Delineation of seed and breeding zones for reforestation and restoration is critical to ensure that seedlings being deployed are optimally adapted to local environmental conditions. In the past, provenance trials were used to achieve this objective. Here we offer an alternative landscape genomic approach based on a machine learning method (gradient forest) to delineate seed and breeding zones. We found that the GF model was able to select environment‐associated (probably adaptive) SNPs for model building. We also found that the six GF‐based seed and breeding zones were comparable to the existing breeding zones of the species and also aligned closely with the major biogeoclimatic zones in BC. With the gradient forest model, we confirmed winter‐related climate variables (MCMT, EMT and DD < 0) are the major climatic factors driving genomic patterns of lodgepole pine local adaptation, although moisture deficits also have a signature. Thus, we concluded that this method can be used to group similarly adapted populations for management purposes. Genotypes from such zones may no longer be planted only within those zones as climates change, but the genetic material from each zone can be treated as similarly adapted when deployed, for example in assisted gene flow. Although this research focused only on lodgepole pine in a specific geographical context, results show advantages over common garden experiments by using genomic information in a gradient forest model to guide the delineation of seed and breeding zones for other widely spread species.

## Author contributions

TW and SNA conceived the idea; YY performed the literature review, conducted modelling and statistical analysis and made the graphics with supporting advice from TW; YY and TW wrote the manuscript; and LHR and SNA edited and improved it.

## Supporting information


**Fig. S1** Split‐density graph of all 20 climate variables involved in the full gradient forest model.
**Fig. S2** Split‐density graph of all 20 climate variables involved in the candidate gradient forest model.
**Fig. S3** Cumulative importance curves for all 20 climate variables involved in the full gradient forest model.
**Fig. S4** Cumulative importance curves for all 20 climate variables involved in the candidate gradient forest model.
**Fig. S5** Principal components analysis biplot of the full and the candidate gradient forest‐predicted genomic variation for British Columbia and Alberta, Canada.
**Fig. S6** Elevation plot and regional names for British Columbia and Alberta, Canada.
**Fig. S7** Four and nine seed and breeding zone maps generated by the full and candidate gradient forest‐model.
**Methods S1** Description of the gradient forest model‐fitting process.
**Table S1** Predictor importance for 20 climate variables generated by the full and the candidate gradient forest model.
**Table S2** Within‐cluster variation and reduction in within‐cluster variation when a different number of clusters was applied to the full and the candidate model predicted continuous genomic variation for lodgepole pine.
**Table S3** Backward comparison between the two sets of common garden‐based seed and breeding zones delineated by Liepe *et al*. (2016; nine zones) and by Ukrainetz *et al*. (2018; four zones) with the corresponding nine‐ and four‐seed and breeding zones delineated based on the full and the candidate models.
**Table S4** Abbreviations and characteristics of biogeoclimatic ecological zones in British Columbia, Canada.
Please note: Wiley Blackwell are not responsible for the content or functionality of any Supporting Information supplied by the authors. Any queries (other than missing material) should be directed to the *New Phytologist* Central Office.

## Data Availability

Lodgepole pine SNPs data are archived at doi: 10.5061/dryad.56j8vq8 (MacLachlan, 2019; Mahony *et al*., 2020). Code for this publication is archived at: https://github.com/yueyu27/gradient‐forest‐for‐delineating‐seed‐and‐breeding‐zones.
