# Supplementary material for: Using landscape genomics to delineate seed and breeding zones for lodgepole pine
Source: New Phytol. 2022 Jun 13;235(4):1653–64. doi: 10.1111/nph.18223 (PMC9545436; doi:10.1111/nph.18223)
Supplement: Supplementary file 1 — Fig. S1 Split‐density graph of all 20 climate variables involved in the full gradient forest model. Fig. S2 Split‐density graph of all 20 climate variables involved in the candidate gradient forest model. Fig. S3 Cumulative importance curves for all 20 climate variables involved in the full gradient forest model. Fig. S4 Cumulative importance curves for all 20 climate variables involved in the candidate gradient forest model. Fig. S5 Principal components analysis biplot of the full and the candidate gradient forest‐predicted genomic variation for British Columbia and Alberta, Canada. Fig. S6 Elevation plot and regional names for British Columbia and Alberta, Canada. Fig. S7 Four and nine seed and breeding zone maps generated by the full and candidate gradient forest‐model. Methods S1 Description of the gradient forest model‐fitting process. Table S1 Predictor importance for 20 climate variables generated by the full and the candidate gradient forest model. Table S2 Within‐cluster variation and reduction in within‐cluster variation when a different number of clusters was applied to the full and the candidate model predicted continuous genomic variation for lodgepole pine. Table S3 Backward comparison between the two sets of common garden‐based seed and breeding zones delineated by Liepe et al. (2016; nine zones) and by Ukrainetz et al. (2018; four zones) with the corresponding nine‐ and four‐seed and breeding zones delineated based on the full and the candidate models. Table S4 Abbreviations and characteristics of biogeoclimatic ecological zones in British Columbia, Canada. Please note: Wiley Blackwell are not responsible for the content or functionality of any Supporting Information supplied by the authors. Any queries (other than missing material) should be directed to the New Phytologist Central Office. [file NPH-235-1653-s001.pdf]

New Phytologist

***New Phytologist* Supporting Information**

Article title: Using landscape genomics to delineate seed and breeding zones for lodgepole pine
Authors: Yue Yu, Sally N. Aitken, Loren H. Rieseberg, Tongli Wang
Article acceptance date: 8 May 2022

The following Supporting Information is available for this article:

Fig. S1 Split density graph of all 20 climate variables involved in the full gradient forest model.

Fig. S2 Split density graph of all 20 climate variables involved in the candidate gradient forest model.

Fig. S3 Cumulative importance curves for all 20 climate variables involved in the full gradient forest model.

Fig. S4 Cumulative importance curves for all 20 climate variables involved in the candidate gradient forest model.

Fig. S5 Principal components analysis biplot of the full and the candidate gradient forest (GF)-predicted genomic variation for British Columbia and Alberta, Canada.

Fig. S6 Elevation plot and regional names for British Columbia and Alberta, Canada.

Fig. S7 Four and nine seed and breeding zone maps generated by the full and candidate gradient forest (GF)-model.

Table S1 Predictor importance for 20 climate variables generated by the full and the candidate gradient forest model.

Table S2 Within-cluster variation and reduction in within-cluster variation when a different number of clusters was applied to the full and the candidate model predicted continuous genomic variation for lodgepole pine.

Table S3 Backward comparison between the two sets of common-garden based seed and breeding zones delineated by Liepe et al. (2016; nine zones) and by Ukrainetz et al. (2018; four zones) with the corresponding nine and four seed and breeding zones delineated based on the full and the candidate models for lodgepole pine in western Canada (see Fig. S7). Liepe et al. delineated British Columbia and Alberta into nine zones, while Ukrainetz et al. had four zones for British Columbia. Average overlap rates were calculated as the percentage of the areas in a

zone that overlapped.

Table S4 Abbreviations and characteristics of biogeoclimatic ecological zones in British Columbia, Canada.

Methods S1 Description of the gradient forest model fitting process.

Fig. S1 Split density graph of all 20 climate variables involved in the full gradient forest model. See Table 1 for descriptions of climate variable abbreviations. Grey bars show the binned raw importance density generated by the random forest output. The black line shows raw importance density I(x). The red line indicates the density of data d(x). The blue line is the estimated importance f(x) = I(x)/d(x). The dashed horizontal line marks where the f(x) ratio is at one (Ellis *et al.*, 2012).

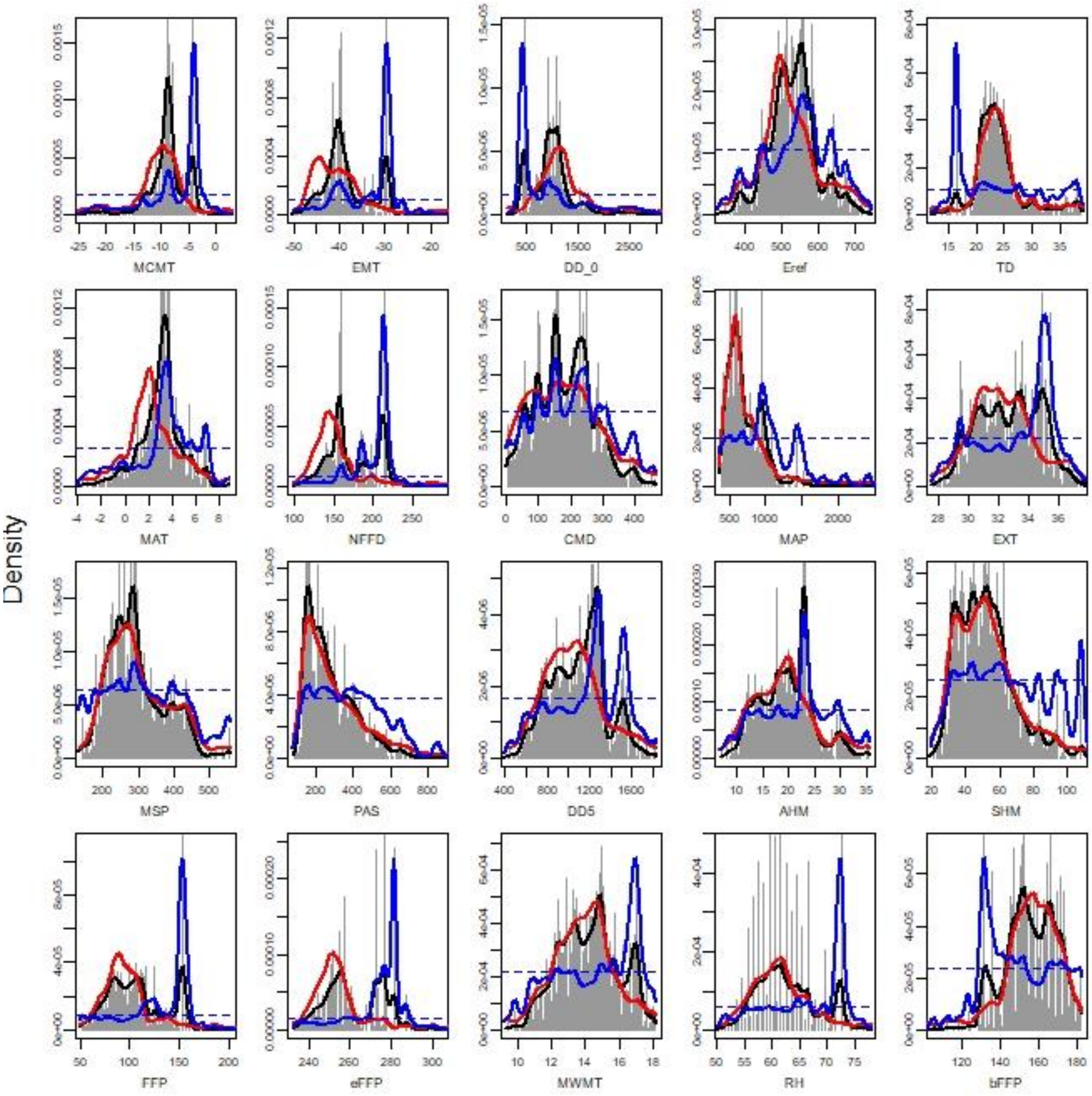

Fig. S2 Split density graph of all 20 climate variables involved in the candidate gradient forest model. See Table 1 for descriptions of climate variable abbreviations. Grey bars show the binned raw importance density generated by the random forest output. The black line shows raw importance density I(x). The red line indicates the density of data d(x). The blue line is the estimated importance f(x) = I(x)/d(x). The dashed horizontal line marks where the f(x) ratio is at one (Ellis *et al*., 2012).

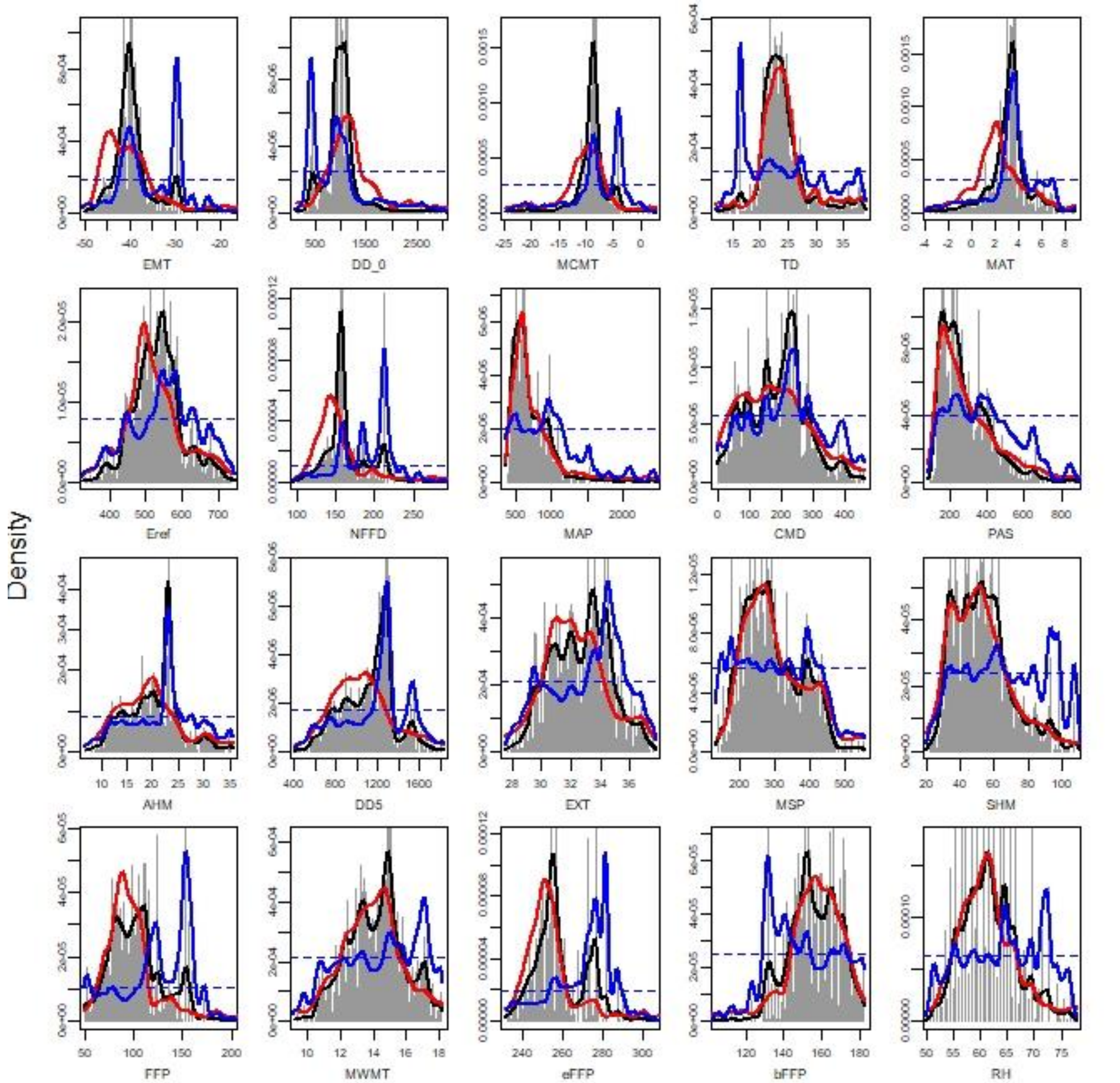

Fig. S3 Cumulative importance curves for all 20 climate variables involved in the full gradient forest model. See Table 1 for descriptions of climate variable abbreviations.

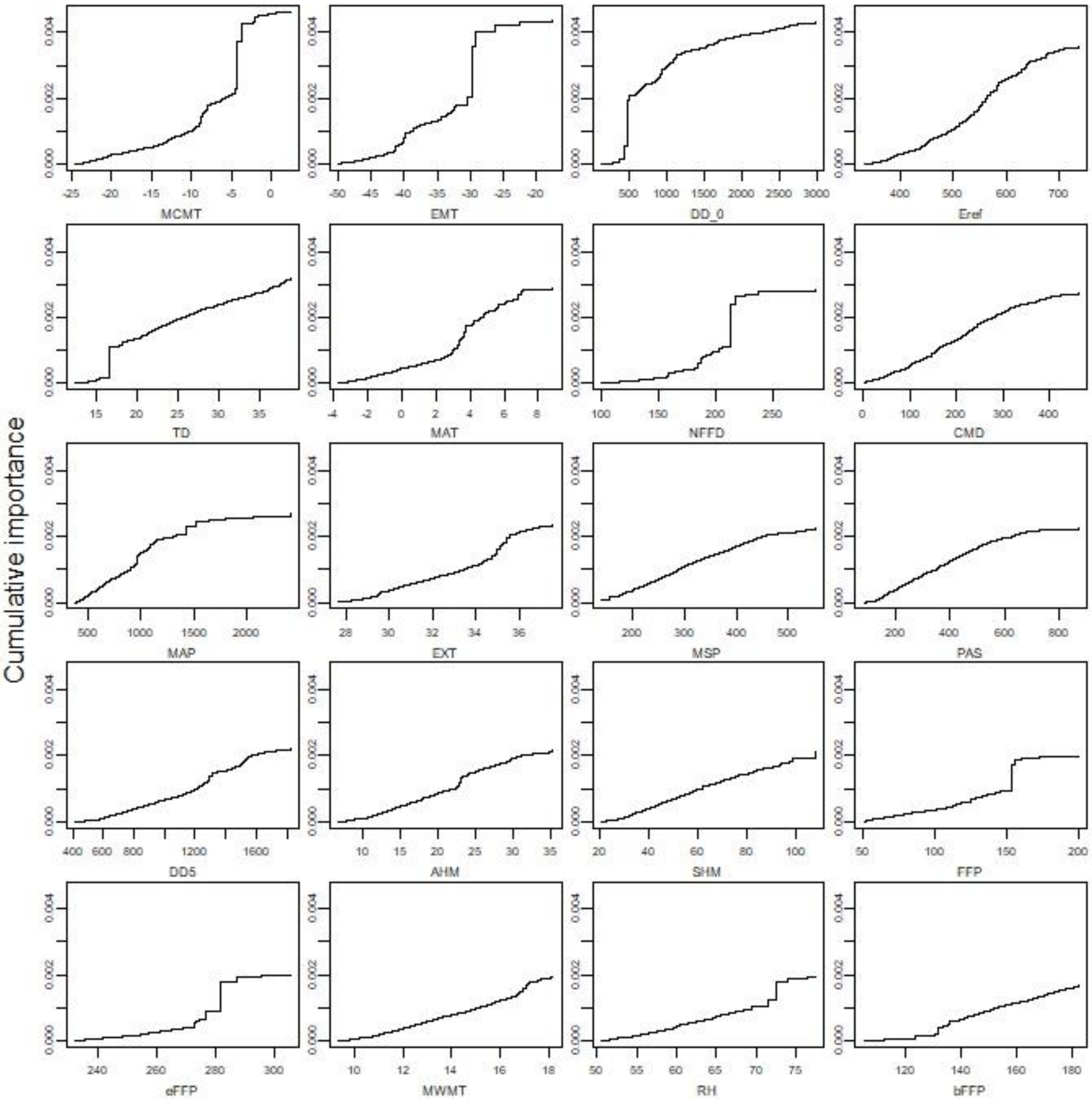

Fig. S4 Cumulative importance curves for all 20 climate variables involved in the candidate gradient forest model. See Table 1 for descriptions of climate variable abbreviations.

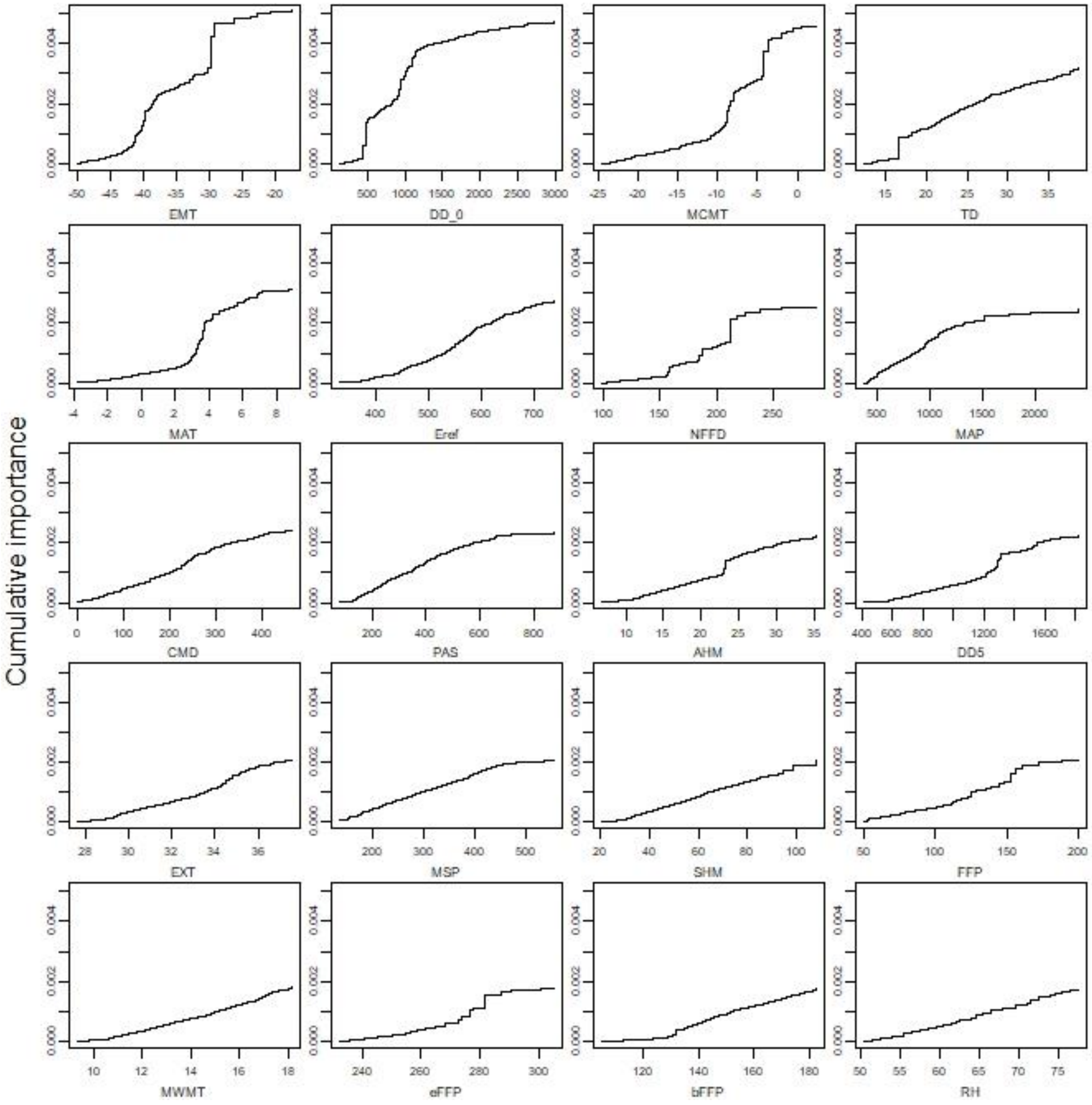

Fig. S5 Principal components analysis biplot of the full (a) and the candidate (b) gradient forest (GF)-predicted genomic variation for British Columbia and Alberta, Canada. Each point in the biplot represents the principal component (PC) score for locations in the lodgepole pine distribution range in British Columbia and Alberta, Canada. See Table 1 for descriptions of climate variable abbreviations.

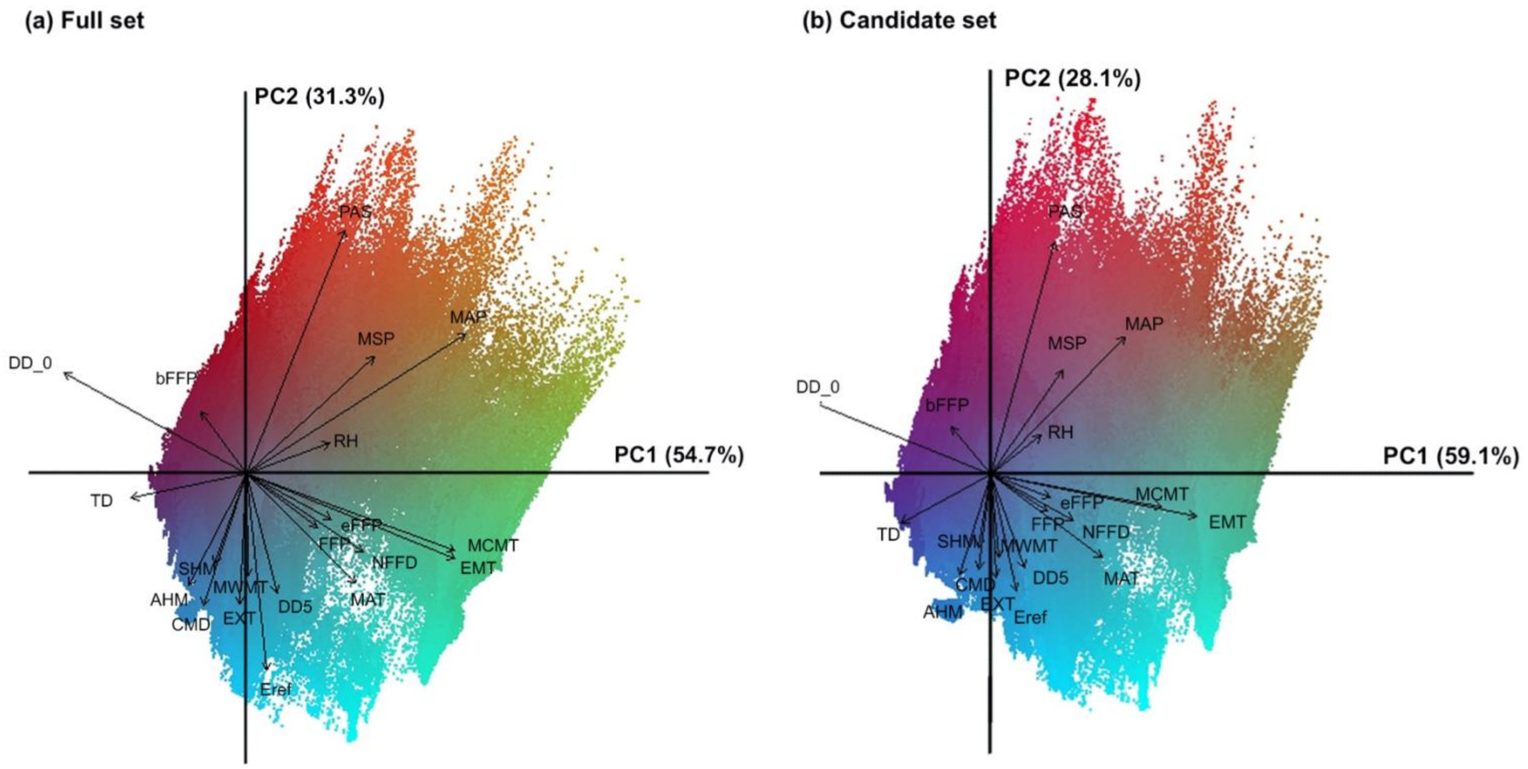

Fig. S6 Elevation plot and regional names for British Columbia and Alberta, Canada.

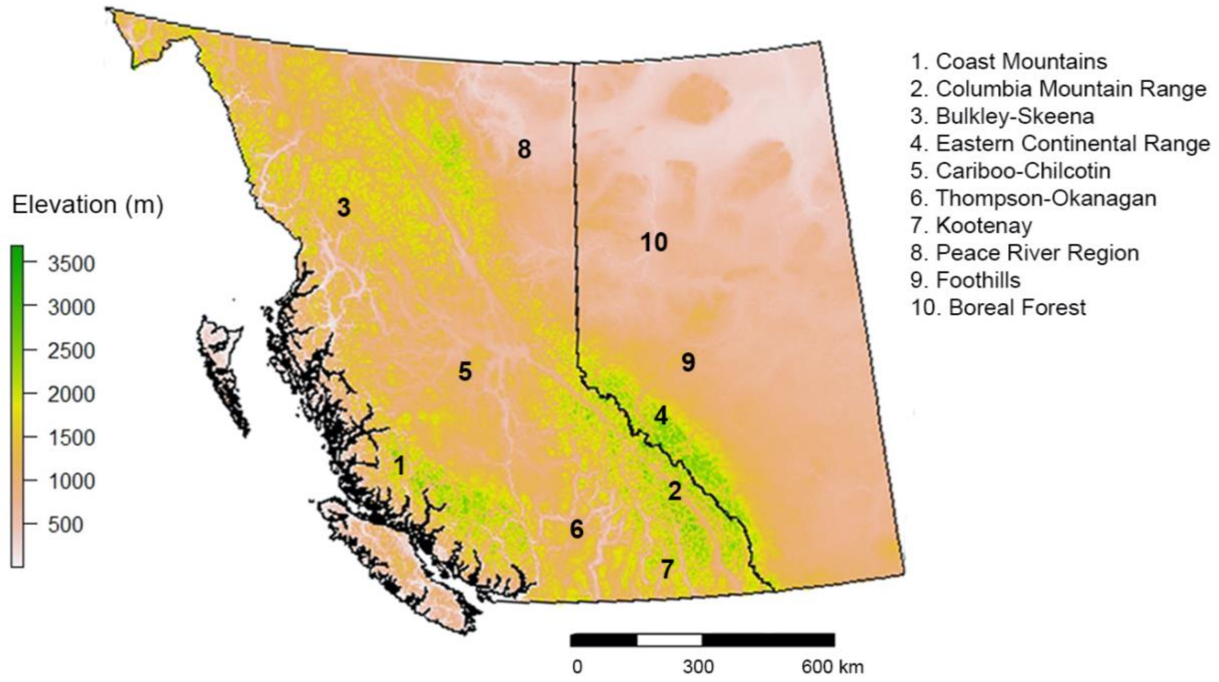

Fig. S7 Four and nine seed and breeding zone maps generated by the full and candidate gradient forest (GF)-model. (a) Four zones by the full GF-model. (b) Nine zones by the full GF-model. (c) Four zones by the candidate GF-model. (d) Nine zones by the candidate GF-model.

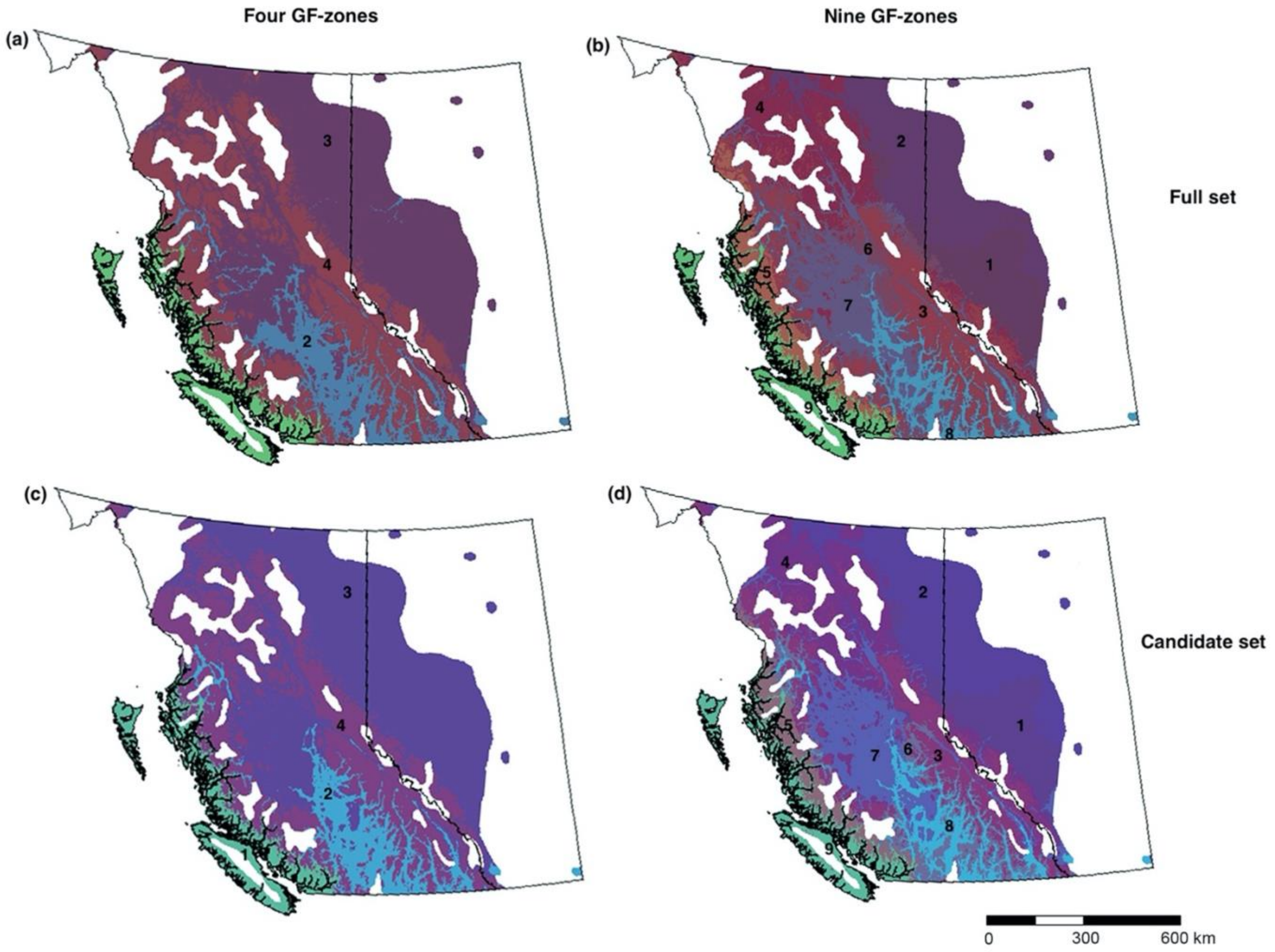

Table S1 Predictor importance for 20 climate variables generated by the full and the candidate gradient forest model. See Table 1 for descriptions of climate variable abbreviations.

Climate variable	Full set		Candidate set	
	Rank	Predictor importance	Rank	Predictor importance
MCMT	1	0.0046	3	0.0046
EMT	2	0.0043	1	0.0051
DD_0	3	0.0043	2	0.0047
Eref	4	0.0036	6	0.0027
TD	5	0.0032	4	0.0032
MAT	6	0.0029	5	0.0031
NFFD	7	0.0028	7	0.0025
CMD	8	0.0027	9	0.0024
MAP	9	0.0027	8	0.0025
EXT	10	0.0023	13	0.0021
MSP	11	0.0023	14	0.0021
PAS	12	0.0023	10	0.0024
DD5	13	0.0022	12	0.0022
AHM	14	0.0022	11	0.0022
SHM	15	0.0021	15	0.0021
FFP	16	0.0020	16	0.0020
eFFP	17	0.0020	18	0.0018
MWMT	18	0.0019	17	0.0018
RH	19	0.0019	20	0.0017
bFFP	20	0.0017	19	0.0017

Table S2 Within-cluster variation and reduction in within-cluster variation when a different number of clusters was applied to the full and the candidate model predicted continuous genomic variation for lodgepole pine.

Number of clusters	Full set		Candidate set	
	**Within - cluster variation**	**Reduction in within-cluster variation**	**Within - cluster variation**	**Reduction in within-cluster variation**
2	0.078	NA*	0.077	NA*
3	0.053	0.0253	0.056	0.0206
4	0.044	0.0091	0.045	0.0114
5	0.036	0.0077	0.038	0.0067
6	0.031	0.0051	0.034	0.0048
7	0.029	0.0025	0.030	0.0033
8	0.027	0.0022	0.028	0.0025
9	0.025	0.0015	0.026	0.0022
10	0.024	0.0012	0.024	0.0017
11	0.023	0.0012	0.023	0.0014
12	0.021	0.0012	0.021	0.0012
13	0.020	0.0010	0.020	0.0009
14	0.020	0.0008	0.019	0.0011
15	0.019	0.0008	0.019	0.0008
16	0.018	0.0007	0.018	0.0006

*NA: Not applicable

Table S3 Backward comparison between the two sets of common-garden based seed and breeding zones delineated by Liepe *et al*. (2016; nine zones) and by Ukrainetz *et al*. (2018; four zones) with the corresponding nine and four seed and breeding zones delineated based on the full and the candidate models for lodgepole pine in western Canada (see Fig. S7). Liepe *et al*. delineated British Columbia and Alberta into nine zones, while Ukrainetz *et al*. had four zones for British Columbia. Average overlap rates were calculated as the percentage of the areas in a zone that overlapped.

Common garden-based zones	GF-based zone number	Common garden-based zone (zone number)	Full set			Candidate set		
			Overlap rate (%)	Overlapping area (km^2)	Averaged overlap rate (%)	Overlap rate (%)	Overlapping area (km^2)	Averaged overlap rate (%)
Liepe *et al*. (2016) zones	1	Montane AB (1)	31.8	32,217	49.5	34.5	34,970	53.5
	2	Lower Foothills (2)	53.5	76,873		59.5	85,415	
	3	Montane BC (3)	40.9	19,008		33.0	15,309	
	4	Lower Boreal Highlands AB (4)	18.7	14,017		23.8	17,834	
	5	Dry Mixed Wood AB (5)	0.0	0		0.0	0	
	6	Sub-boreal (6)	28.5	27,217		11.6	11,086	
	7	Sub-boreal (7)	62.3	50,516		68.4	55,432	
	8	Interior Valleys (8)	65.4	23,198		65.6	23,259	
	9	Coastal BC (9)	72.8	17,880		73.0	17,933	
Ukrainetz *et al*. (2018) zones	1	Breeding group 1	26.3	68,229	56.8	28.3	73,654	57.4
	2	Breeding group 2	49.3	72,171		48.4	70,824	
	3	Breeding group 3	68.8	165,127		73.5	176,338	
	4	Breeding group 4	59.3	261,046		57.3	252,127	

Table S4 Abbreviations and characteristics of biogeoclimatic ecological zones in British Columbia, Canada (all values are averaged; Wang *et al*., 2012).

BEC zone	Abbreviation	Latitude N(°)	Longitude W(°)	Elevation (m)	MAT (°C)	MAP (mm)	CONT (°)
Boreal Altai Fescue Alpine	BAFA	57.49	128.66	1685	-2.5	1101	22.5
Bunchgrass	BG	50.73	121.11	610	5.9	342	23.8
Boreal White and Black Spruce	BWBS	58.17	123.88	719	-0.3	514	30.3
Coastal Douglas-fir	CDF	49.04	123.71	73	9.5	1092	13.9
Coastal Mountain-heather Alpine	CMA	54.02	128.60	1561	0.0	3197	19.2
Coastal Western Hemlock	CWH	51.61	127.01	418	6.5	2900	15.0
Engelmann Spruce–Subalpine Fir	ESSF	53.39	122.30	1552	0.3	1103	22.1
Interior Cedar–Hemlock	ICH	51.99	120.61	977	3.2	919	23.0
Interior Douglas-fir	IDF	50.84	120.89	1019	3.9	493	22.8
Interior Mountain-heather Alpine	IMA	51.61	119.01	2261	-1.6	1570	20.6
Mountain Hemlock	MH	52.78	127.29	1065	2.9	3114	17.7
Montane Spruce	MS	50.85	120.70	1438	1.8	649	22.0
Ponderosa Pine	PP	49.88	119.07	643	6.4	379	23.8
Sub-Boreal Pine–Spruce	SBPS	52.41	123.86	1152	1.7	472	22.8
Sub-Boreal Spruce	SBS	54.35	124.33	900	2.2	656	23.9
Spruce–Willow–Birch	SWB	58.44	128.23	1293	-1.8	691	24.7

Methods S1 Description of the gradient forest (GF) model fitting process (Ellis et al., 2012; Fitzpatrick & Keller, 2015).

Gradient forest (GF) is based on an aggregation of random forests. It builds a random forest for each of the SNPs with the goodness-of-fit for each SNP being presented as R^2. Only SNPs with R^2 values above zero were considered to be associated with the predictors (having a positive predictive power) and were employed to build the final GF model. For each of the GF-selected SNPs, splits in every regression tree of the random forest are dependent on the smallest impurity (sum of the squared deviation of group mean). The importance of each split, representing the amount of variation explained by that split, was recorded as the impurity importance (also known as raw importance). The impurity importance for each SNP along each predictor gradient generated an impurity importance bar graph, which was then placed in order in an array. This array contained information for all GF-selected SNPs and all predictors included in model fitting. The impurity importance was then aggregated and averaged among all GF-selected SNPs for each predictor to provide the information for the overall predictor importance.

Raw importance gathered across all GF-selected SNPs for each predictor was then combined into the split importance graph using kernel density estimation. The split importance graph, including the information on binned raw importance and a kernel density estimation of the binned bars, was named the raw importance density. The raw importance density curve was then scaled by data density, resulting in the estimated importance curve, which was further normalized to ensure the area under the estimated importance curve for each predictor sums up to the predictor importance. Finally, cumulative importance curves for each predictor were generated by taking the integral of the estimated importance. Information from the cumulative importance curves converted environmental gradients into genomic gradients in multidimensional space, which serve as the basis for the model prediction.

References

Ellis N, Smith SJ, Pitcher CR. 2012. Gradient forests: calculating importance gradients on physical predictors. *Ecology* **93**: 156-168. http://www.jstor.org/stable/23144030.

Fitzpatrick MC, Keller SR. 2015. Ecological genomics meets community-level modelling of biodiversity: mapping the genomic landscape of current and future environmental adaptation. *Ecology Letters* **18**: 1-16. https://doi.org/10.1111/ele.12376

Liepe KJ, Hamann A, Smets P, Fitzpatrick CR, Aitken SN. 2016. Adaptation of lodgepole pine and interior spruce to climate: implications for reforestation in a warming world. *Evolutionary applications* **9**: 409-419. https://doi.org/10.1111/eva.12345

Ukrainetz NK, Yanchuk AD, Mansfield SD. 2018. Climatic drivers of genotype–environment interactions in lodgepole pine based on multi-environment trial data and a factor analytic model of additive covariance. *Canadian Journal of Forest Research* **48**: 835-854. https://doi.org/10.1139/cjfr-2017-0367

Wang T, Campbell EM, O'Neill GA, Aitken SN. 2012. Projecting future distributions of ecosystem climate niches: uncertainties and management applications. *Forest Ecology and Management* **279**:128-140. https://doi.org/10.1016/j.foreco.2012.05.034
